# Preimplantation genetic testing for aneuploidy: challenges in clinical practice

**DOI:** 10.1186/s40246-022-00442-8

**Published:** 2022-12-20

**Authors:** Hui Yang, Andrew Thomas DeWan, Mayur M. Desai, Sten H. Vermund

**Affiliations:** 1 Yale School of Public Health, Advanced Professional MPH Program, 60 College Street, New Haven, CT 06510 USA; 2 Yale Center for Perinatal, Pediatric and Environmental Epidemiology, Chronic Disease Epidemiology, Yale School of Public Health, 1 Church Street, Fl 6Th Floor, New Haven, CT 06510 USA; 3 Yale School of Public Health, 60 College Street, PO Box 208034, New Haven, CT 06520-8034 USA; 4 Department of Pediatrics, Yale School of Medicine, New Haven, CT 06510 USA

**Keywords:** Preimplantation genetic testing, Pregnancy, In vitro fertilization, Aneuploidy, Mosaicism, Screening, Ethics

## Abstract

Preimplantation genetic testing for aneuploidy (PGT-A) has been used widely during in vitro fertilization procedures in assisted reproductive centers throughout the world. Despite its wide use, concerns arise from the use of PGT-A technology in clinical decision-making. We address knowledge gaps in PGT-A, summarizing major challenges and current professional guidelines. First, PGT-A is a screening test and not a diagnostic test. Second, mosaicism is much higher in the blastocyst stage from PGT-A than had been recognized previously and a mosaic embryo may not accurately represent the genetic disease risk for future fetal disorders. Third, PGT-A was not validated clinically before use in patients; the best use of this technology for selected age-groups remains uncertain. Given these gaps, we believe that current professional policies relying on industry-self-regulation are insufficient. In the USA, the Food and Drug Administration may be the most appropriate agency to provide more definitive guidelines and regulations that are needed for better practice.

## Background

### Use of preimplantation genetic testing

Preimplantation genetic testing (PGT) is used to genetically evaluate embryos before transfer to the uterus during in vitro fertilization (IVF) procedures [1]. Genetic tests are performed on DNA obtained from biopsied cells from early embryonic stages [2]. There are three different types of PGT: (1) for aneuploidy (PGT-A), such as Down Syndrome and Turner Syndrome; (2) for monogenic/single gene defects (PGT-M), such as myotonic dystrophy and cystic fibrosis; (3) and for chromosomal structural rearrangements (PGT-SR) [1]. PGT-SR includes reciprocal and Robertsonian translocations, insertional translocations, deletions, duplications, and inversions. The benefits of PGT-M and PGT-SR are well accepted [3]. However, the outcome benefits of PGT-A have been more controversial. PGT-A has become a routine add-on for IVF to improve clinical outcomes worldwide. However, the lack of clinical validation and high false-positive rate are extremely concerning [4].

The world’s first PGT procedure was conducted in 1989 for a patient with an X-linked disease [2]. Polymerase chain reaction (PCR) technology was used to test for a Y chromosome to identify the sex of the embryo in order to select and transfer only female embryos [2]. PGT is a relatively complicated procedure because it requires a biopsy from the embryo, during which minimal harm to the embryo must be ensured. In the past 20 years, different versions of PGT-A have been introduced into the IVF clinic. In the 1990s and early 2000s, fluorescence in situ hybridization (FISH) was used but it can only detect a reduced number of chromosomes [5]. It was known as preimplantation genetic screening (PGS) 1.0, which was later shown to have no outcome benefit [6]. In 2008, technological innovation and changes in biopsy procedures led to PGS 2.0, which is the most common currently applied version of PGT-A.

More recently, genome-wide array platforms have started to become more widely utilized in PGT-A leading to PGS 3.0. These include array comparative genomic hybridization (aCGH), single nucleotide polymorphisms (SNP) arrays and next-generation sequencing (NGS) [5]. In July 2016, the Preimplantation Genetics Diagnosis International Society (PGDIS) published the first guidance for PGS and changed the procedure name from PGS to PGT-A [4]. Currently, the genetic testing industry is working on what could be called PGS 4.0, using cell-free DNA from the cultured embryos created in IVF [4].

By the late twentieth century, PGT was used to screen for a few serious genetic diseases with a high incidence rate in the sampled populations, such as Tay-Sachs and cystic fibrosis [7]. With the development of new, more affordable technologies, PGT-A has been expanded as a screening test for parents of advanced maternal age undergoing IVF in an attempt to increase clinical success rates and reduce the chance of a variety of genetic diseases in the offspring [1]. Aside from the technical challenges of PGT-A, the impact of PGT-A on pregnancy outcomes as well as ethical and moral concerns are at the forefront of debates considering the use of PGT-A technology. In this commentary, we seek to address whether selecting an embryo based on the PGT-A method is realistic considering the existing challenges of PGT-A and potential limitations in making a decision to discard an embryo.

## Gaps in Knowledge

After the introduction of genetic screening, many patients did not understand the limitations of the different types of PGT [8]. For instance, the effectiveness and/or clinical benefit of PGT-A as a screening test for IVF patients has not yet been fully evaluated [4]. A screening test is the testing of unaffected people to find those at increased risk of having a disease or disorder. From a cost-effectiveness perspective, routinely adding PGT-A should not be recommended [9–11]. As of late 2022, there is no established diagnostic test that can be applied during early embryological stages to confirm the PGT-A results. Two recent non-selection studies have been performed to estimate predictive value and reproductive outcome for PGT-A [12, 13], showing promising predictive values.

Another area of uncertainty is that mosaicism is much higher in preimplantation human embryos than initially expected by clinical researchers [14]. Mosaicism indicates that more than two types of genetically different sets of cells are present in an embryo. Scientists are still trying to understand mosaicism in PGT-A [15], but a mosaic embryo could lead to a healthy baby. Several studies have shown that the transfer of mosaicism embryos can result in non-notable healthy babies [16]. One explanation is that the mosaic embryo can undergo self-correction during differentiation and proliferation [16]. This means that the biopsied cell from PGT-A may not accurately represent the embryo and thus lead to a false-positive or false-negative result. Despite these advanced scientific technologies, more work is needed to develop evidence-based classification systems to accurately select the mosaic embryos [17]. For example, Viotti et al. [18] showed that mosaic blastocysts have poorer clinical outcomes compared to the euploid group (57.2% vs. 46.5% for successful implantation) and there was a correlation between the number of mosaic chromosomes and unfavorable outcomes. However, Viotti et al. [18] reported that in 94.6% of cases, a mosaic embryo was selected for transfer because no euploid embryos were available, suggesting the importance of a scoring system to prioritize mosaic embryos. Another concern is that the mosaicism rate has been influenced by the stage of biopsy and analytical methods [5], making it challenging to predict offspring outcomes from the diagnosis of chromosomal mosaicism in a trophectoderm biopsy [19, 20]. Other barriers include the lack of prospective studies evaluating the long-term outcome of the children or an evidence-based system for prioritizing embryos according to the risk stratification results [5]. Therefore, the best method to use for predicting the offspring outcome from preimplantation mosaicism data remains undetermined [15, 20]. Non-selection trials can provide unbiased population selection processes. Information on chromosomal abnormalities, including uniform and mosaic aneuploidies, can introduce predictive values to evaluate the likelihood of aneuploidy/mosaicism of PGT-A [21].

An opinion article published by reproductive medicine experts suggests finding a better term than “embryo mosaic” [22]. They suggest using a more accurate term: “intermediate copy number,” because microarray and NGS profile cannot diagnose chromosomal mosaicism in a trophectoderm biopsy. This test was never clinically validated, nor certified by a regulatory agency or professional organization before it became a routine test in IVF clinics [4].

What does this mean for a patient undergoing IVF? After exhausting and expensive IVF and PGT procedures, patients have to face another challenge in deciding the fate of the embryo based on uncertain, and/or inaccurate results.

Finally, it is critical to consider which group of patients can benefit from PGT-A. Roche et al. [23] used data from the Society for Assisted Reproductive Technology (SART) to evaluate the use of PGT-A in the USA. They found that PGT-A use increased sharply in the years 2014 to 2017, though live birth rates did not increase [23]. A systematic review summarized 11 randomized controlled trials (RCTs) of PGT-A and found that PGT-A improved the live birth rate of women over 35 years old, but it did not improve the outcomes of the general population [24]. Theobald et al. [25] conducted a cross-sectional study that showed 32.6% of PGT-A screens were performed in women under 35 years old in 2016. These studies demonstrate that PGT-A was broadly used in the general population instead of a targeted population, hindering robust conclusions regarding the efficiency of PGT-A. Although RCTs are important, abnormal embryos are not typically transferred or followed up in those studies [21]. Therefore, RCTs cannot address an essential question which is how many embryos are falsely discarded as aneuploid, highlighting the importance of non-selection studies prior to clinical implementation of PGT-A.

All this uncertainty can leave patients confused [26]. During IVF, patients are confronted with a plethora of questions that will undoubtedly affect their lives as well as the fate of their embryos: Should we test or not test? Should we transfer a mosaic embryo or not despite the result indicating a potential genetic disorder? Should we accept the high risk of a genetic disease based on these mosaicism results, and forgot IVF and opt for adoption?

### What are the current professional recommendations?

Several professional associations have updated their guidelines for PGT-A [15, 27, 28]. The American College of Obstetricians and Gynecologists (ACOG) 2020 guidelines state that there is no clear evidence to use PGT-A routinely, and the best use of PGT-A still needs to be determined [27]. The ACOG 2020 guideline also emphasizes that negative PGT-A results will not guarantee a baby without genetic abnormalities [27]. The Practice Committee of the American Society of Reproductive Medicine and the Society for Assisted Reproductive Technology has a similar recommendation: the value of the PGT-A as a screening test for IVF requires further investigation [28]. The Practice Committee and Genetic Counseling Professional Group (GCPG) of the American Society for Reproductive Medicine 2020 committee opinion does not endorse or suggest that PGT-A is appropriate for all cases of IVF [15]. The GCPG conclusion claims that there is no evidence-based classification system to guide embryo selection at this stage [15].

Where does this leave the patient? PGT-A is a tool for use by clinicians to prioritize the order of transferring embryos. Patients may not wish to engage in embryo selection in most cases, unless they are highly motivated and well-informed. PGT-A was widely used in many IVF centers in the USA before clinicians realized the limitations of this technology [4]. One comparison study showed that the use of PGT in the UK remained consistent at under 2% from 2014 to 2016; however, the usage of PGT in the USA increased from 13 to 27% during the same time period [25]. The cost of PGT-A in the UK is approximately $4000 (£3000), but in the USA it can be as high as $12,000 [25]. In the UK, 40% of assisted reproductive technology is funded by the National Health Service but does not cover PGT-A [25]. In contrast, the majority of assisted reproductive technology in the USA is self-funded and with little regulatory oversight from the government or federal organizations [25].

For optimal patient care, further rigorous clinical validation is needed for PGT-A. IVF is a multibillion-dollar business, and the industry has introduced many advanced techniques without proper clinical validation [2, 4]. After performing PGT-A on thousands of IVF patients, several committees stated that the value of PGT-A testing remains to be determined. When a new technology is introduced into clinical practice, proper preclinical evaluation of the method is needed. This has not been the case so far with PGT-A. It is common for many IVF patients to have already experienced several failures of pregnancy and they may be willing to try new methods without necessarily considering or understanding the cost–benefit. This can also be the result of a lack of understanding and/or consensus among health professionals in accurately educating and informing patients regarding relatively new medical procedures such as PGT-A. This new technology was made widely available prior to demonstrating evidence-based results confirming the efficacy of PGT-A.

It remains a challenge as to exactly how to best educate patients. The Centers for Disease Control and Prevention (CDC) requires that patient education materials be written at or below a fifth-grade reading level [29]. Early et al. conducted an environmental scan of IVF and PGT patient education materials and found that among the 17 sets of educational materials examined, none of these materials met the CDC standard [29]. These findings suggest that patient PGT educational materials may not always be comprehensible or clear to all patients [29]; comprehension is influenced by the range of educational backgrounds and familiarity with medical terminology and concepts. Lack of appropriate educational materials that present information in an accessible, unbiased, and comprehensible manner have the potential to lead to disparities in the utilization of PGT, primarily because patient educational materials have exceeded the average literacy skills of US residents. Another factor is the extent to which healthcare providers reviewed PGT educational materials with patients during IVF visits, such as offering a concise summary of procedures, pros/cons, discussion, and time for addressing patient any questions (genetics counseling discussed below).

### Is professional self-regulation effective in the USA?

Unlike many European countries that have legislation regarding the use of PGT-A [30], PGT-A is merely regulated by professional guidelines in the USA, referred to as “professional self-regulation.” This means that a fertility specialist can refer their patients to perform any genetic testing available in the market [7]. Because the USA is a permissive country, medical recommendations vary among different clinics and physicians. Parental selection of fetal sex is allowed, which also attracts some patients to travel to the USA from countries that have “hard” restrictions (e.g., China) to select the sex of the embryo. In the USA, a “soft” policy allows individual clinicians to decide under which conditions to use PGT and where to send the samples, which can include a laboratory that does not necessarily possess Clinical Laboratory Improvement Amendments (CLIA) certification. Many patients may not realize their rights and these legal requirements (i.e., PGT-A testing conducted in a lab that is CLIA-certified).

Although professional guidelines encourage or require genetic counseling, few counties require it by legislative mandate [1]. The USA does not require genetic counseling for all uses of PGT but requires that a woman seeking PGT be informed and advised [31]. However, there are no clear professional guidelines. Insufficient genetic counseling is a concern in IVF clinics. The European Society of Human Reproduction and Embryology (ESHRE) PGT Consortium provides very detailed recommendations about patient inclusion and exclusion criteria for the decision to accept or decline patients in PGT services, counseling and patient follow-up procedures [1]. ESHRE also provides technical recommendations for each test, regulations and laboratory requirements [32–34]. Such recommendations can ensure that PGT patients receive the best care possible.

## Conclusions

With the advancement in reproductive technologies, it is critical to establish regulation in the USA to assure proper preclinical evaluation and continuous quality assessment of PGT-A services. The current professional self-regulation system for PGT-A may not be sufficient. A designated agency may be needed to monitor PGT-A use and address relevant concerns. For example, the FDA could provide oversight related to the accuracy of results and mosaicism, the indication of PGT-A as a medical necessity vs for personal and social reasons, and provide guidelines for developing and implementing patient education. Oversight from a designated federal agency would include detailed guidance for the selection of appropriate new procedures, methodologies, and laboratory requirements, reducing uncertainty as to the risks and benefits of the procedure [35]. Additionally, health professional organizations need to produce clear guidelines before a new test can be used in the clinic. Evidence-based data are also needed to evaluate the risk and benefit for patients. Finally, IVF clinics should provide adequate genetic counseling to all patients [36]. Educational materials should be easily understandable and readable for the general population. Improved communication between medical professionals and patients can also help provide support for patients grappling with these challenging decisions regarding their embryos.

## Data Availability

Data sharing is not applicable to this article as no datasets were generated or analyzed during the current study.

## Abbreviations

ACOG: The American College of Obstetricians and Gynecologists
CVS: Chorionic villus sampling
CDC: The Centers for Disease Control and Prevention
CLIA: Clinical Laboratory Improvement Amendments
ESHRE: The European Society of Human Reproduction and Embryology
FDA: Food and Drug Administration
GCPG: Genetic Counseling Professional Group
IVF: In vitro fertilization
PGT: Preimplantation genetic testing
PCR: Polymerase chain reaction
PGS: Preimplantation genetic screening
RCTs: Randomized clinical trials
SART: The Society for Assisted Reproductive Technology
